# Crystal structure of 2^10^,2^20^-bis­(2,6-di­chloro­phen­yl)-4,7,12,15-tetra­oxa-2(5,15)-nickel(II)porpyhrina-1,3(1,2)-dibenzena-cyclo­hepta­deca­phane-9-yne di­chloro­methane monosolvate

**DOI:** 10.1107/S2056989019007527

**Published:** 2019-05-31

**Authors:** Morten K. Peters, Christian Näther, Rainer Herges

**Affiliations:** a Otto-Diels-Institut für Organische Chemie, Christian-Albrechts-Universität Kiel, Otto-Hahn-Platz 4, D-24098 Kiel, Germany; b Institut für Anorganische Chemie, Christian-Albrechts-Universität Kiel, Max-Eyth Str. 2, D-24118 Kiel, Germany

**Keywords:** crystal structure, Ni^II^ porphyrine, hydrogen bonding, twinning, pseudo-symmetry

## Abstract

The asymmetric unit of the title compound, [Ni(C_52_H_34_Cl_4_N_4_O_4_)]·CH_2_Cl_2_, consists of two discrete complexes, which show significant differences in the conformation of the side chain. Each Ni^II^ cation is coordinated by four nitro­gen atoms of a porphyrin mol­ecule within a square-planar coordination environment. Weak intra­molecular C—H⋯Cl and C—H⋯O inter­actions stabilize the mol­ecular conformation. In the crystal structure, discrete complexes are linked by C—H⋯Cl hydrogen-bonding inter­actions. In addition, the two unique di­chloro­methane solvate mol­ecules (one being disordered) are hydrogen-bonded to the Cl atoms of the chloro­phenyl groups of the porphyrin mol­ecules, thus stabilizing the three-dimensional arrangement. The crystal exhibits pseudo-ortho­rhom­bic metrics, but structure refinements clearly show that the crystal system is monoclinic and that the crystal is twinned by pseudo-merohedry.

## Abstract 2

In the crystal structure of the title compound, the Ni^II^ cations in the two unique complexes are coordinated in a square-planar coordination environment by the N atoms of a porphyrin mol­ecule.

## Chemical context   

The crystal structures of several strapped (Peters *et al.*, 2019 ▸), capped (Ganesh & Sanders, 1980 ▸), hindered (Momenteau *et al.*, 1983 ▸) and bridged porphyrins (Battersby & Hamilton, 1980 ▸) have been determined. Strapped porphyrins are of extraordinary importance because they exhibit different structural features, which allow a wide range of applications (Goncalves & Sanders, 2007 ▸) and have been used as chiral epoxidation catalysts (Collman *et al.*, 1995 ▸), as models for enzymes such as cytochrome P450 (Andrioletti *et al.*, 1999 ▸), as building blocks for the synthesis of catenanes (Gunter *et al.*, 1994 ▸), as building blocks for self-assembled photonic wires (Koepf *et al.*, 2005 ▸), or as models for a number of biomimetic porphyrins (Morgan & Dolphin, 1987 ▸).

**Figure 1:**
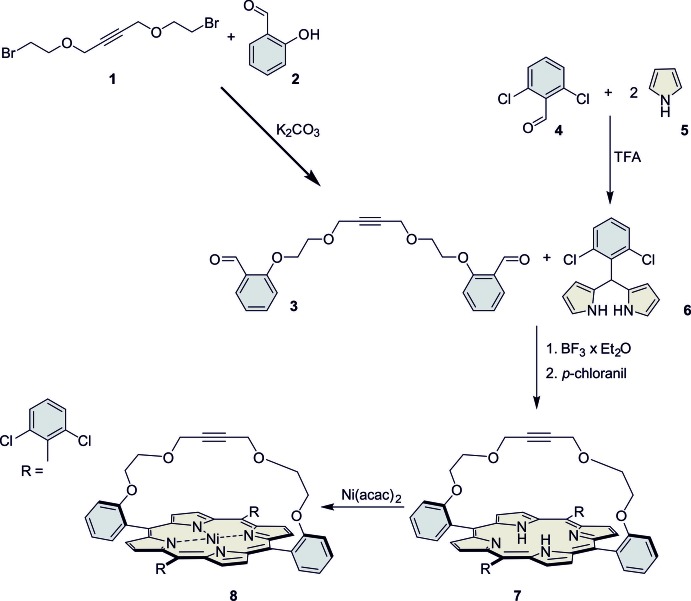
Reaction scheme for the synthesis of the title compound.

In our ongoing investigations on this topic, we became inter­ested in the synthesis of the title compound, which was prepared by the following strategy, as detailed in the reaction scheme (Fig. 1 ▸): salicyl­aldehyde (**2**) and 1,4-bis­(2-bromo­eth­oxy)-2-butyne (**1**) were reacted to give 2,2′-({[but-2-yne-1,4-diylbis(­oxy)]bis­(ethane-2,1-di­yl)}bis­(­oxy))dibenzaldehyde (**3**) (Shankar *et al.*, 2018 ▸). The bridge **3** was used in Lindsay-type cyclization reactions with *meso*-(di­chloro­phen­yl)dipyrro­methane (**6**) (Littler *et al.*, 1999 ▸) to afford strapped porphyrins with yields of up to 14%. Upon heating a solution of the free-base porphyrin (**7**) with nickel(II) acetyl­acetonate in toluene to 383 K, the title Ni^II^-porphyrin (**8**) was obtained in 80% yield. We inserted Ni^II^ into the porphyrin because nickel-hydro­porphyrins are powerful catalysts in reduction processes in nature, and in technologically important reactions (Peters & Herges, 2018 ▸). Furthermore, Ni^II^-porphyrins have been used as responsive contrast agents in functional magnetic resonance imaging (*f*MRI) (Venkataramani *et al.*, 2011 ▸; Dommaschk *et al.*, 2015*a* ▸,*b* ▸; Peters *et al.*, 2018 ▸). The reaction product was crystallized from a di­chloro­methane solution and was unambiguously characterized by single crystal X-ray diffraction.
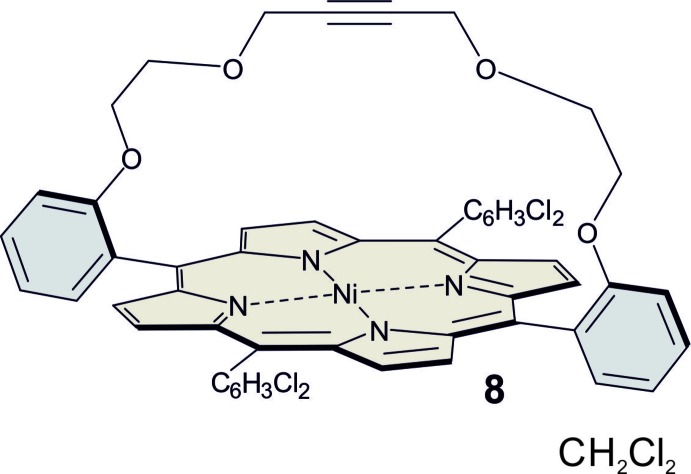


## Structural commentary   

**Figure 2:**
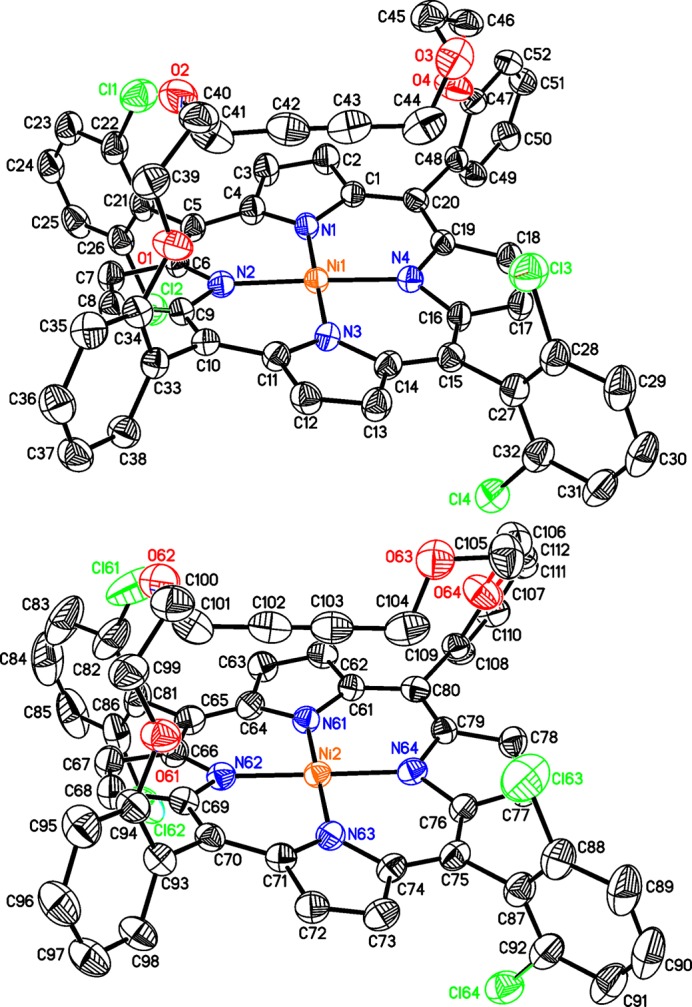
Mol­ecular structures of the two crystallographically independent complexes and solvent mol­ecules with the atom labelling and displacement ellipsoids drawn at the 50% probability level. For clarity, the H atoms and the solvent molecules have been omitted.

**Figure 3:**
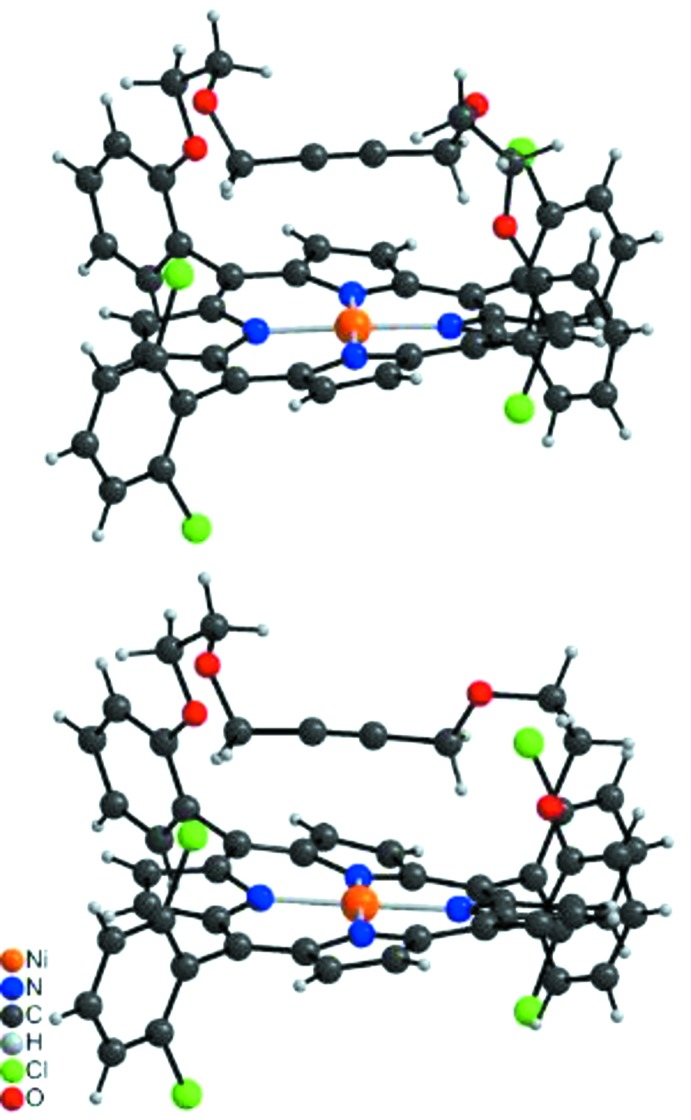
Side view of the two crystallographically independent complexes, showing the conformational differences in the side chains.

**Figure 4:**
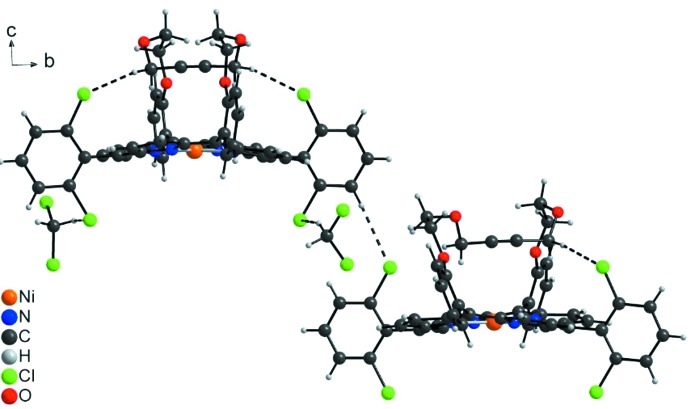
Crystal structure of the title compound showing intra- and inter­molecular C—H⋯Cl hydrogen bonding as dashed lines. The disorder of one of the two crystallographically independent solvent mol­ecules is not shown for clarity.

The crystal structure of the title compound consists of discrete Ni-porphyrin complexes, in which the Ni^II^ cations show a square-planar coordination (Fig. 2 ▸). The asymmetric unit consists of two complexes in general positions that show a significantly different conformation in their bridging side chain (Fig. 3 ▸). The Ni—N bond lengths are similar in both complexes and range from 1.937 (2) to 1.950 (3) Å (Table 1 ▸), in accordance with literature values (Liu *et al.*, 2016 ▸). In both complexes, the Ni^II^ cations are situated in the porphyrin ring plane (Fig. 3 ▸), with root-mean-square deviations of 0.0276 Å for mol­ecule 1 (Ni1) and of 0.0186 Å for mol­ecule 2 (Ni2). The 2,6-di­chloro­phenyl groups are nearly perpendicular to the corresponding porphyrin planes with dihedral angles of 89.82 (4) and 88.23 (4)° (mol­ecule 1) and 88.89 (5) and 85.82 (4)° (mol­ecule 2). This conformation is consolidated by intra­molecular C—H⋯Cl hydrogen bonding between the methyl­ene groups of the side chains and the Cl atoms of the 2,6-dichlorphenyl rings (Fig. 4 ▸, Table 2 ▸). In addition, the conformation of each side chain is stabilized by intra­molecular C—H⋯O bonding (Table 2 ▸).

The asymmetric unit additionally contains two di­chloro­methane mol­ecules in general positions, one of which is disordered (Fig. 2 ▸).

## Supra­molecular features   

**Figure 5:**
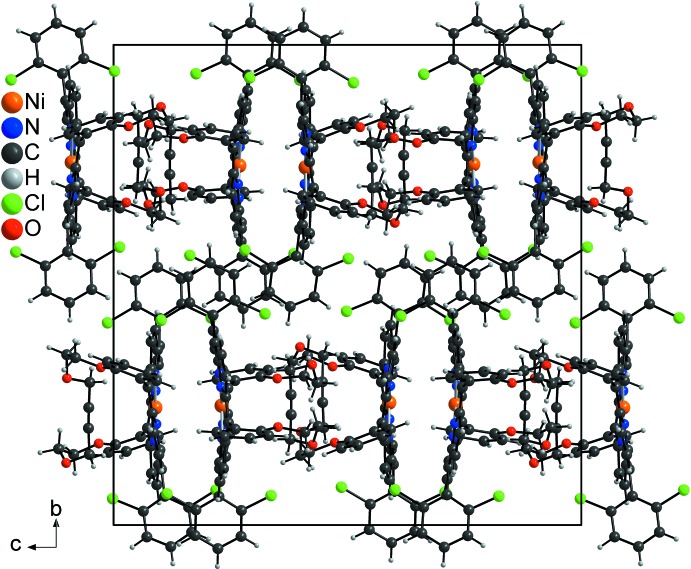
Crystal structure of the title compound in a view along the *a* axis. The solvent mol­ecules are omitted for clarity.

In the crystal structure, the porphyrine ring planes are aligned parallel to the *ab* plane and are shifted along the *a* axis, whereas the 2,6-di­chloro­phenyl substitutents are arranged in layers parallel to the *ac* plane (Fig. 5 ▸). Within these planes, the dichlormethane solvate mol­ecules are embedded and are linked to the Cl atoms of the complexes by weak inter­molecular C—H⋯Cl hydrogen bonding (Fig. 4 ▸), thus stabilizing the three-dimensional arrangement.

## Database survey   

According to a search in the Cambridge Structural Database (CSD, version 5.40, updated Feb. 2019; Groom *et al.*, 2016 ▸), 790 structures with nickel porphyrins have been deposited. This includes six similar strapped nickel(II) porphyrins: (5,15-{2,2′-[pentane-1,5-diylbis(­oxy)]bis­(5-*t*-butyl­phen­yl)}-10,20-bis­(4-nitro­phen­yl)porphyrinato)nickel(II) (Liu *et al.*, 2016 ▸), (5,15-{2,2′-[pro­pane-1,3-diylbis(­oxy)]bis­(5-*t*-butyl­phen­yl)}10,20-bis­(4-nitro­phen­yl)porphyrinato)nickel(II) (Liu *et al.*, 2016 ▸), (5,15-{2,2′-[butane-1,4-diylbis(­oxy)]bis­(5-*t*-butyl­phen­yl)}10,20-bis­(4-nitro­phen­yl)porphyrinato)nickel(II) (Liu *et al.*, 2016 ▸), (5,15-{2,2′-[hexane-1,6-diylbis(­oxy)]bis­(5-*t*-butyl­phen­yl)}-10,20-bis(4-nitro­phen­yl)porphyrinato)nickel(II) (Liu *et al.*, 2016 ▸) (5,15-{2,2′-[heptane-1,7-diylbis(­oxy)]bis­(5-*t*-butyl­phen­yl)}-10,20-bis(4-nitro­phen­yl)porphyrinato)nickel(II) (Liu *et al.*, 2016 ▸) and (4,19-di-*t*-butyl-11,12,45,46-tetra­methyl-8,15-dioxa-41,42,43,44-tetra-aza­nona­cyclo­[20.9.9.2^10,13^.1^23,26^.1^28,31^.1^32,35^.1^37,40^.0^2,7^.0^16,21^]hexa­tetra­conta-1(31),2,4,6,10,12,16,18,20,22,24,26,28(43),29,32,34,36,38,40,45-icosa­enato)nick­el(II) (Gehrold *et al.*, 2015 ▸). Furthermore, strapped iron (Sabat & Ibers, 1982 ▸), zinc (Gunter *et al.*, 2004 ▸) and copper porphyrins (Liu *et al.*, 2016 ▸) have also been reported.

## Synthesis and crystallization   

**Synthesis**

The general synthesis scheme is given in Fig. 1 ▸. 1,4-Bis(2-bromo­eth­oxy)-2-butyne (**1**), *meso*-di­chloro­phenyl dipyrro­methane (**6**) and 2,2′-({[but-2-yne-1,4-diylbis(­oxy)]bis­(ethane-2,1-di­yl)}bis­(­oxy))dibenzaldehyde (**3**) were synthesized as reported (Shankar *et al.*, 2018 ▸; Littler *et al.*, 1999 ▸).

**Synthesis of 2^10^,2^20^-bis­(2,6-di­chloro­phen­yl)-4,7,12,15-tetra­oxa-2(5,15)-porpyhrina-1,3(1,2)-dibenzena-cyclo­hepta­deca­phane-9-yn**e **(7)**

2,2′-({[But-2-yne-1,4-diylbis(­oxy)]bis­(ethane-2,1-di­yl)}bis(­oxy))dibenzaldehyde (**3**) (375 mg, 983 µmol) and boron trifluoride etherate (13.9 mg, 98.3 µmol) were dissolved in di­chloro­methane (350 ml) under a nitro­gen atmosphere. To this solution *meso*-di­chloro­phenyl dipyrro­methane (436 mg, 1.96 mmol), dissolved in di­chloro­methane (50 ml), was added under stirring over a period of 1 h. After further stirring for 15 h, *p*-chloranil (504 mg, 2.05 mmol) was added and stirred for 5 h at 313 K. Then the solvent was removed under reduced pressure and the crude product was purified by column chromatography (di­chloro­methane, *R_f_* = 0.07). A purple solid was obtained (129 mg, 140 µmol, 14%); m.p. 400 K; ^1^H NMR (500 MHz, CDCl_3_, 300 K): *δ* = 8.79 (*d*, *^3^J* = 4.5 Hz, 4H), 8.61 (*d*, *^3^J* = 4.5 Hz, 4H), 8.54 (*d*, *^3^J* = 6.8 Hz, 2H), 7.82 (*dd*, *^3^J* = 8.1 Hz, *^4^J* = 1.2 Hz, 2H), 7.77–7.66 (*m*, 6H), 7.50 (*t*, *^3^J* = 7.4 Hz, 2H), 7.07 (*d*, *^3^J* = 8.0 Hz, 2H), 3.69 (*s*, *br*, 4H), 2.46 (*s*, *br*, 4H), 0.89 (*m*, 4H), −2.52 (*s*, *br*, 2H, NH) ppm; HRMS (EI): 920.14750 (calculated). 920.14750 (found) for C_52_H_36_Cl_4_N_4_O_4_.

**Synthesis of 2^10^,2^20^-bis­(2,6-di­chloro­phen­yl)-4,7,12,15-tetra­oxa-2(5,15)-nickel(II)porpyhrina-1,3(1,2)-dibenzena-cyclo­hepta­deca­phane-9-yne (8)**

5,15-Strapped porphyrin (**7**) (13.0 mg, 14.1 µmol) and nickel(II) acetyl­acetonate (182 mg, 707 µmol) were dissolved in toluene (100 ml) and stirred under reflux for 4 d. The solvent was removed under reduced pressure and the crude product was purified by column chromatography (di­chloro­methane, *R_f_* = 0.14). A purple solid was obtained (11.0 mg, 11.3 mmol, 80%); m.p. 612 K; ^1^H NMR (500 MHz, CD_2_Cl_2_, 300 K): *δ* = 8.79 (*d*, *^3^J* = 4.9 Hz, 4H), 8.57 (*d*, *^3^J* = 4.9 Hz, 4H), 8.46 (*dd*, *^3^J* = 7.3 Hz, *^4^J* = 1.7 Hz, 2H), 7.88 (*dd*, *^3^J* = 8.2 Hz, *^4^J* = 1.2 Hz, 2H), 7.71 (*td*, *^3^J* = 8.1 Hz, *^4^J* = 1.7 Hz, 2H), 7.67 (*t*, *^3^J* = 8.2 Hz, 2H), 7.63 (*dd*, *^3^J* = 8.2 Hz, *^4^J* = 1.2 Hz, 2H), 7.50 (*td*, *^3^J* = 7.6 Hz, *^4^J* = 0.9 Hz, 2H), 7.08 (*d*, *^3^J* = 8.3 Hz, 2H), 3.79 (*t*, *^3^J* = 4.2 Hz, 4H), 2.80 (*t*, *^3^J* = 4.2 Hz, 4H), 1.70 (*s*, 4H) ppm; HRMS (EI): 976.06620 (calculated). 976.06876 (found) for C_52_H_34_Cl_4_N_4_NiO_4_.

**Crystallization**

The layering technique was used for crystallization of the title compound. The lower layer consisted of (**8**) dissolved in di­chloro­methane, and for the upper layer *n*-heptane was used.

## Refinement   

Crystal data, data collection and structure refinement details are summarized in Table 3 ▸.

The crystal metrics points to ortho­rhom­bic symmetry with the inter­nal *R*-value only slightly higher in the ortho­rhom­bic system compared to the monoclinic system. Additionally, the ADDSYM option in *PLATON* (Spek, 2009 ▸) indicates a higher (pseudo)-symmetry for the monoclinic solution with 85% fit and missing *n* and *c*-glide planes, with *Pccn* as the most probable space group. Structure solution in *Pccn* led to two crystallographically independent mol­ecules in the asymmetric unit that are each located on a twofold rotation axis. However, the acetyl­ene side chain of one of these mol­ecules is completely disordered around this axis, which indicates that the crystal symmetry is too high. Moreover, structure refinement in *Pccn* led to very poor reliability factors with *wR*_2_ values of about 50%, revealing that the true symmetry is in fact monoclinic. Therefore the structure was refined in the monoclinic space group *P*2_1_/*c* under consideration of twinning by pseudo-merohedry (mirror plane parallel to *ab* as twin element), which resulted in two crystallographically independent and fully ordered mol­ecules, much better reliability factors and a BASF parameter of 0.5895 (8).

The C—H hydrogen atoms were positioned with idealized geometries (C—H = 0.95–0.99 Å) and were refined with *U*_iso_(H) = 1.2*U*_eq_(C) using a riding model. One of the two crystallographically independent di­chloro­methane mol­ecules is equally disordered and was refined with a split model using restraints for the bond lengths and for components of the anisotropic displacement parameters. 

## Supplementary Material

Crystal structure: contains datablock(s) I. DOI: 10.1107/S2056989019007527/wm5502sup1.cif

Structure factors: contains datablock(s) I. DOI: 10.1107/S2056989019007527/wm5502Isup2.hkl

CCDC reference: 1918135

Additional supporting information:  crystallographic information; 3D view; checkCIF report

## Figures and Tables

**Table 1:** Selected geometric parameters (Å, °)

Ni1—N4	1.937 (2)	Ni2—N63	1.937 (3)
Ni1—N2	1.942 (2)	Ni2—N64	1.939 (3)
Ni1—N1	1.943 (3)	Ni2—N62	1.948 (3)
Ni1—N3	1.946 (3)	Ni2—N61	1.950 (3)
			
N4—Ni1—N2	177.92 (9)	N63—Ni2—N64	90.11 (10)
N4—Ni1—N1	90.12 (10)	N63—Ni2—N62	89.96 (11)
N2—Ni1—N1	89.82 (10)	N64—Ni2—N62	179.09 (9)
N4—Ni1—N3	89.77 (10)	N63—Ni2—N61	178.44 (9)
N2—Ni1—N3	90.35 (10)	N64—Ni2—N61	89.59 (10)
N1—Ni1—N3	178.43 (9)	N62—Ni2—N61	90.37 (10)

**Table 2:** Hydrogen-bond geometry (Å, °)

*D*—H⋯*A*	*D*—H	H⋯*A*	*D*⋯*A*	*D*—H⋯*A*
C3—H3⋯Cl62^i^	0.95	2.86	3.566 (4)	132
C13—H13⋯Cl64	0.95	2.89	3.632 (3)	136
C31—H31⋯Cl63^ii^	0.95	2.95	3.878 (4)	165
C41—H41*A*⋯Cl1	0.99	2.94	3.918 (4)	169
C41—H41*B*⋯O1	0.99	2.39	3.037 (5)	122
C44—H44*A*⋯Cl3	0.99	2.91	3.867 (4)	163
C44—H44*B*⋯O4	0.99	2.37	3.029 (5)	123
C63—H63⋯Cl2^iii^	0.95	2.87	3.669 (3)	142
C73—H73⋯Cl4	0.95	2.83	3.639 (3)	143
C101—H10*C*⋯Cl61	0.99	2.75	3.734 (4)	172
C101—H10*D*⋯O61	0.99	2.30	2.962 (4)	123
C104—H10*F*⋯N64	0.99	2.67	3.410 (5)	132
C104—H10*F*⋯O64	0.99	2.40	3.028 (5)	121
C121—H20*B*⋯O62^iv^	0.99	2.65	3.304 (7)	124
C121—H20*A*⋯Cl2^v^	0.99	2.90	3.563 (6)	125
C122—H20*F*⋯Cl4^iv^	0.99	2.70	3.583 (6)	149

Symmetry codes: (i) 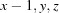; (ii) 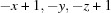; (iii) 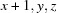; (iv) 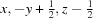; (v) 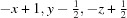.

**Table 3:** Experimental details

Crystal data	
Chemical formula	[Ni(C_52_H_34_Cl_4_N_4_O_4_)]·CH_2_Cl_2_
*M*_r_	1064.27
Crystal system, space group	Monoclinic, *P*2_1_/*c*
Temperature (K)	170
*a*, *b*, *c* (Å)	15.4185 (3), 24.9658 (4), 24.3053 (5)
β (°)	90.039 (2)
*V* (Å^3^)	9356.0 (3)
*Z*	8
Radiation type	Mo *K*α
μ (mm^−1^)	0.81
Crystal size (mm)	0.2 × 0.1 × 0.1

Data collection	
Diffractometer	STOE IPDS2
Absorption correction	Numerical (*X-RED* and *X-SHAPE*; Stoe, 2008 ▸)
*T*_min_, *T*_max_	0.761, 0.956
No. of measured, independent and observed [*I* > 2σ(*I*)] reflections	40366, 17160, 14957
*R*_int_	0.031
(sin θ/λ)_max_ (Å^−1^)	0.617

Refinement	
*R*[*F* ^2^ > 2σ(*F* ^2^)], *wR*(*F* ^2^), *S*	0.040, 0.106, 1.05
No. of reflections	17160
No. of parameters	1235
No. of restraints	11
H-atom treatment	H-atom parameters constrained
Δρ_max_, Δρ_min_ (e Å^−3^)	0.33, −0.47

Computer programs: *X-AREA* (Stoe, 2008 ▸), *SHELXT* (Sheldrick, 2015*a* ▸), *SHELXL2014* (Sheldrick, 2015*b* ▸), *XP* in *SHELXTL* (Sheldrick, 2008 ▸), *DIAMOND* (Brandenburg, 2014 ▸) and *publCIF* (Westrip, 2010 ▸).

## supplementary crystallographic information

### Crystal data

**Table:**

[Ni(C_52_H_34_Cl_4_N_4_O_4_)]·CH_2_Cl_2_	*F*(000) = 4352
*M**_r_* = 1064.27	*D*_x_ = 1.511 Mg m^−^^3^
Monoclinic, *P*2_1_/*c*	Mo *K*α radiation, λ = 0.71073 Å
*a* = 15.4185 (3) Å	Cell parameters from 41412 reflections
*b* = 24.9658 (4) Å	θ = 1.3–26.3°
*c* = 24.3053 (5) Å	µ = 0.81 mm^−^^1^
β = 90.039 (2)°	*T* = 170 K
*V* = 9356.0 (3) Å^3^	Block, colorless
*Z* = 8	0.2 × 0.1 × 0.1 mm

### Data collection

**Table:**

STOE IPDS-2 diffractometer	14957 reflections with *I* > 2σ(*I*)
ω scans	*R*_int_ = 0.031
Absorption correction: numerical (X-RED and X-SHAPE; Stoe, 2008)	θ_max_ = 26.0°, θ_min_ = 1.3°
*T*_min_ = 0.761, *T*_max_ = 0.956	*h* = −19→18
40366 measured reflections	*k* = −24→30
17160 independent reflections	*l* = −29→29

### Refinement

**Table:**

Refinement on *F*^2^	11 restraints
Least-squares matrix: full	Hydrogen site location: inferred from neighbouring sites
*R*[*F*^2^ > 2σ(*F*^2^)] = 0.040	H-atom parameters constrained
*wR*(*F*^2^) = 0.106	*w* = 1/[σ^2^(*F*_o_^2^) + (0.0618*P*)^2^ + 2.2115*P*] where *P* = (*F*_o_^2^ + 2*F*_c_^2^)/3
*S* = 1.05	(Δ/σ)_max_ = 0.001
17160 reflections	Δρ_max_ = 0.33 e Å^−^^3^
1235 parameters	Δρ_min_ = −0.47 e Å^−^^3^

### Special details

**Table:**

Geometry. All esds (except the esd in the dihedral angle between two l.s. planes) are estimated using the full covariance matrix. The cell ESDS are taken into account individually in the estimation of esds in distances, angles and torsion angles; correlations between esds in cell parameters are only used when they are defined by crystal symmetry. An approximate (isotropic) treatment of cell esds is used for estimating esds involving l.s. planes.
Refinement. Refined as a two-component twin

### Fractional atomic coordinates and isotropic or equivalent isotropic displacement parameters (Å^2^)

**Table:**

	*x*	*y*	*z*	*U*_iso_*/*U*_eq_	Occ. (<1)
Ni1	0.24928 (3)	0.25134 (2)	0.26878 (2)	0.02680 (7)	
N1	0.14001 (16)	0.28997 (9)	0.26571 (10)	0.0302 (5)	
N2	0.31155 (16)	0.31894 (10)	0.26754 (10)	0.0302 (5)	
N3	0.35862 (17)	0.21240 (9)	0.26968 (10)	0.0308 (5)	
N4	0.18696 (16)	0.18408 (9)	0.27290 (10)	0.0285 (5)	
C1	0.05825 (19)	0.26855 (12)	0.25630 (12)	0.0308 (6)	
C2	−0.0048 (2)	0.31073 (13)	0.25185 (16)	0.0411 (8)	
H2	−0.0649	0.3065	0.2443	0.049*	
C3	0.0370 (2)	0.35723 (13)	0.26038 (16)	0.0428 (8)	
H3	0.0116	0.3919	0.2613	0.051*	
C4	0.1273 (2)	0.34470 (12)	0.26785 (14)	0.0354 (7)	
C5	0.1908 (2)	0.38252 (12)	0.27332 (13)	0.0332 (6)	
C6	0.2782 (2)	0.36993 (11)	0.27325 (13)	0.0311 (6)	
C7	0.3457 (2)	0.40930 (12)	0.27256 (13)	0.0367 (7)	
H7	0.3392	0.4469	0.2767	0.044*	
C8	0.4204 (2)	0.38231 (12)	0.26489 (13)	0.0354 (7)	
H8	0.4767	0.3976	0.2622	0.043*	
C9	0.4003 (2)	0.32695 (12)	0.26163 (12)	0.0296 (6)	
C10	0.4611 (2)	0.28666 (11)	0.25454 (11)	0.0293 (6)	
C11	0.44087 (19)	0.23311 (12)	0.26037 (12)	0.0300 (6)	
C12	0.5036 (2)	0.19079 (12)	0.26001 (13)	0.0347 (7)	
H12	0.5641	0.1946	0.2539	0.042*	
C13	0.4616 (2)	0.14506 (12)	0.26979 (14)	0.0369 (7)	
H13	0.4868	0.1104	0.2725	0.044*	
C14	0.3714 (2)	0.15796 (11)	0.27553 (12)	0.0315 (6)	
C15	0.3073 (2)	0.12056 (12)	0.28372 (12)	0.0320 (6)	
C16	0.2203 (2)	0.13335 (12)	0.28290 (13)	0.0319 (6)	
C17	0.1521 (2)	0.09519 (12)	0.28682 (14)	0.0388 (7)	
H17	0.1584	0.0582	0.2953	0.047*	
C18	0.0771 (2)	0.12098 (13)	0.27631 (14)	0.0383 (7)	
H18	0.0209	0.1054	0.2751	0.046*	
C19	0.09797 (19)	0.17605 (11)	0.26736 (12)	0.0289 (6)	
C20	0.0380 (2)	0.21514 (12)	0.25569 (12)	0.0301 (6)	
C21	0.1653 (2)	0.44041 (12)	0.27483 (14)	0.0377 (7)	
C22	0.1512 (2)	0.46951 (13)	0.22672 (14)	0.0422 (7)	
C23	0.1272 (2)	0.52349 (14)	0.22740 (17)	0.0491 (9)	
H23	0.1170	0.5422	0.1940	0.059*	
C24	0.1187 (2)	0.54901 (13)	0.27698 (17)	0.0497 (9)	
H24	0.1037	0.5859	0.2779	0.060*	
C25	0.1317 (2)	0.52170 (14)	0.32562 (17)	0.0484 (9)	
H25	0.1259	0.5396	0.3599	0.058*	
C26	0.1533 (2)	0.46822 (13)	0.32392 (14)	0.0412 (7)	
Cl1	0.16340 (8)	0.43857 (4)	0.16324 (4)	0.0563 (2)	
Cl2	0.16756 (6)	0.43497 (4)	0.38603 (4)	0.0505 (2)	
C27	0.3319 (2)	0.06291 (12)	0.28745 (13)	0.0365 (7)	
C28	0.3439 (2)	0.03165 (13)	0.24050 (14)	0.0409 (7)	
C29	0.3660 (2)	−0.02217 (14)	0.24273 (17)	0.0491 (9)	
H29	0.3729	−0.0423	0.2098	0.059*	
C30	0.3777 (3)	−0.04601 (14)	0.29318 (18)	0.0526 (9)	
H30	0.3917	−0.0830	0.2951	0.063*	
Cl3	0.32728 (7)	0.06013 (4)	0.17628 (4)	0.0533 (2)	
Cl4	0.33567 (7)	0.07334 (4)	0.39818 (4)	0.0495 (2)	
C31	0.3694 (3)	−0.01668 (15)	0.34109 (17)	0.0503 (9)	
H31	0.3788	−0.0330	0.3759	0.060*	
C32	0.3469 (2)	0.03720 (13)	0.33737 (14)	0.0411 (7)	
C33	0.5523 (2)	0.30039 (11)	0.24001 (12)	0.0298 (6)	
C34	0.5750 (2)	0.30439 (12)	0.18438 (12)	0.0330 (6)	
C35	0.6602 (2)	0.31347 (13)	0.16862 (13)	0.0389 (7)	
H35	0.6751	0.3157	0.1308	0.047*	
C36	0.7234 (2)	0.31929 (14)	0.20875 (15)	0.0426 (8)	
H36	0.7819	0.3255	0.1982	0.051*	
C37	0.7024 (2)	0.31612 (14)	0.26365 (15)	0.0404 (8)	
H37	0.7461	0.3203	0.2909	0.048*	
C38	0.6166 (2)	0.30675 (13)	0.27920 (13)	0.0370 (7)	
H38	0.6022	0.3047	0.3171	0.044*	
O1	0.50746 (15)	0.29602 (10)	0.14863 (9)	0.0425 (5)	
C39	0.5211 (2)	0.30663 (14)	0.09100 (13)	0.0425 (7)	
H39A	0.5679	0.2835	0.0763	0.051*	
H39B	0.5375	0.3446	0.0853	0.051*	
C40	0.4359 (2)	0.29438 (15)	0.06273 (13)	0.0437 (8)	
H40A	0.4455	0.2933	0.0225	0.052*	
H40B	0.4158	0.2585	0.0744	0.052*	
O2	0.37005 (18)	0.33245 (10)	0.07449 (11)	0.0511 (6)	
C41	0.3192 (3)	0.32122 (17)	0.12233 (17)	0.0572 (10)	
H41A	0.2766	0.3504	0.1274	0.069*	
H41B	0.3579	0.3211	0.1549	0.069*	
C42	0.2724 (2)	0.26985 (19)	0.12020 (15)	0.0516 (9)	
C43	0.2349 (3)	0.22824 (19)	0.12060 (15)	0.0537 (10)	
C44	0.1872 (3)	0.17769 (18)	0.12289 (17)	0.0567 (10)	
H44A	0.2293	0.1482	0.1282	0.068*	
H44B	0.1487	0.1784	0.1554	0.068*	
O3	0.13625 (19)	0.16618 (11)	0.07557 (11)	0.0546 (7)	
C45	0.0686 (3)	0.20342 (16)	0.06519 (14)	0.0501 (9)	
H45A	0.0590	0.2056	0.0250	0.060*	
H45B	0.0874	0.2392	0.0779	0.060*	
C46	−0.0155 (2)	0.18981 (15)	0.09243 (13)	0.0434 (8)	
H46A	−0.0617	0.2145	0.0800	0.052*	
H46B	−0.0329	0.1527	0.0831	0.052*	
O4	−0.00242 (17)	0.19490 (12)	0.15017 (9)	0.0495 (6)	
C47	−0.0711 (2)	0.19042 (13)	0.18528 (13)	0.0345 (7)	
C48	−0.0520 (2)	0.19920 (11)	0.24058 (12)	0.0304 (6)	
C49	−0.1191 (2)	0.19650 (13)	0.27843 (13)	0.0369 (7)	
H49	−0.1071	0.2034	0.3161	0.044*	
C50	−0.2035 (2)	0.18396 (14)	0.26289 (16)	0.0431 (8)	
H50	−0.2484	0.1817	0.2895	0.052*	
C51	−0.2204 (2)	0.17487 (14)	0.20814 (15)	0.0420 (8)	
H51	−0.2779	0.1666	0.1969	0.050*	
C52	−0.1556 (2)	0.17752 (14)	0.16936 (14)	0.0420 (8)	
H52	−0.1683	0.1706	0.1318	0.050*	
Ni2	0.74995 (3)	0.25519 (2)	0.41082 (2)	0.02937 (8)	
N61	0.86334 (16)	0.28973 (10)	0.40890 (10)	0.0322 (5)	
N62	0.69334 (17)	0.32495 (10)	0.41138 (11)	0.0321 (5)	
N63	0.63757 (17)	0.22047 (10)	0.41061 (10)	0.0328 (5)	
N64	0.80643 (16)	0.18579 (10)	0.41152 (11)	0.0319 (5)	
C61	0.9442 (2)	0.26678 (13)	0.41747 (12)	0.0338 (6)	
C62	1.0102 (2)	0.30699 (13)	0.41504 (14)	0.0402 (7)	
H62	1.0705	0.3016	0.4208	0.048*	
C63	0.9715 (2)	0.35343 (13)	0.40313 (15)	0.0408 (7)	
H63	0.9995	0.3868	0.3972	0.049*	
C64	0.8795 (2)	0.34357 (12)	0.40094 (13)	0.0375 (7)	
C65	0.8191 (2)	0.38379 (13)	0.39767 (13)	0.0376 (7)	
C66	0.7308 (2)	0.37489 (12)	0.40373 (13)	0.0365 (7)	
C67	0.6674 (2)	0.41587 (13)	0.40759 (14)	0.0407 (7)	
H67	0.6772	0.4532	0.4029	0.049*	
C68	0.5908 (2)	0.39235 (13)	0.41910 (14)	0.0397 (7)	
H68	0.5367	0.4097	0.4247	0.048*	
C69	0.6074 (2)	0.33578 (12)	0.42124 (12)	0.0314 (6)	
C70	0.5422 (2)	0.29788 (12)	0.42735 (11)	0.0312 (6)	
C71	0.5567 (2)	0.24356 (12)	0.41900 (12)	0.0311 (6)	
C72	0.4895 (2)	0.20382 (13)	0.41669 (13)	0.0365 (7)	
H72	0.4291	0.2096	0.4215	0.044*	
C73	0.5285 (2)	0.15698 (13)	0.40640 (14)	0.0401 (7)	
H73	0.5006	0.1234	0.4014	0.048*	
C74	0.6200 (2)	0.16659 (12)	0.40428 (12)	0.0327 (6)	
C75	0.6808 (2)	0.12639 (12)	0.40166 (13)	0.0360 (7)	
C76	0.7691 (2)	0.13553 (12)	0.40637 (13)	0.0339 (6)	
C77	0.8325 (2)	0.09441 (13)	0.41019 (15)	0.0442 (8)	
H77	0.8226	0.0570	0.4067	0.053*	
C78	0.9089 (2)	0.11825 (13)	0.41956 (14)	0.0403 (7)	
H78	0.9629	0.1006	0.4250	0.048*	
C79	0.8949 (2)	0.17514 (12)	0.42001 (13)	0.0334 (7)	
C80	0.9587 (2)	0.21276 (13)	0.42617 (12)	0.0338 (7)	
C81	0.8505 (2)	0.43988 (13)	0.39072 (17)	0.0479 (8)	
C82	0.8732 (3)	0.47116 (18)	0.4364 (2)	0.0709 (13)	
C83	0.9044 (3)	0.5245 (2)	0.4290 (3)	0.093 (2)	
H83	0.9179	0.5459	0.4602	0.112*	
C84	0.9149 (3)	0.5447 (2)	0.3776 (4)	0.096 (2)	
H84	0.9360	0.5802	0.3730	0.115*	
C85	0.8955 (3)	0.51460 (19)	0.3328 (3)	0.0830 (18)	
H85	0.9038	0.5288	0.2969	0.100*	
C86	0.8634 (2)	0.46299 (15)	0.33940 (19)	0.0545 (10)	
Cl61	0.86279 (11)	0.44509 (7)	0.50187 (6)	0.1077 (6)	
Cl62	0.83801 (7)	0.42722 (5)	0.28087 (4)	0.0621 (3)	
C87	0.6497 (2)	0.06951 (13)	0.40201 (14)	0.0401 (7)	
C88	0.6306 (3)	0.04335 (15)	0.45143 (16)	0.0519 (9)	
C89	0.6025 (3)	−0.00944 (16)	0.4535 (2)	0.0643 (11)	
H89	0.5908	−0.0261	0.4878	0.077*	
C90	0.5920 (3)	−0.03670 (16)	0.4059 (2)	0.0674 (12)	
H90	0.5735	−0.0730	0.4071	0.081*	
C91	0.6075 (3)	−0.01304 (16)	0.35588 (19)	0.0585 (10)	
H91	0.5981	−0.0324	0.3227	0.070*	
C92	0.6371 (2)	0.03964 (14)	0.35427 (16)	0.0463 (8)	
Cl63	0.64172 (10)	0.07876 (5)	0.51284 (4)	0.0785 (4)	
Cl64	0.65929 (7)	0.06863 (4)	0.29109 (4)	0.0528 (2)	
C93	0.4529 (2)	0.31578 (12)	0.44239 (12)	0.0335 (6)	
C94	0.4346 (2)	0.33259 (13)	0.49596 (13)	0.0382 (7)	
C95	0.3510 (2)	0.34853 (15)	0.51069 (14)	0.0480 (8)	
H95	0.3396	0.3608	0.5470	0.058*	
C96	0.2854 (2)	0.34647 (17)	0.47275 (16)	0.0531 (9)	
H96	0.2285	0.3574	0.4829	0.064*	
C97	0.3011 (2)	0.32867 (17)	0.41970 (16)	0.0503 (9)	
H97	0.2550	0.3265	0.3939	0.060*	
C98	0.3843 (2)	0.31399 (15)	0.40444 (14)	0.0435 (8)	
H98	0.3951	0.3025	0.3678	0.052*	
O61	0.50317 (16)	0.33018 (10)	0.53182 (9)	0.0435 (5)	
C99	0.4936 (3)	0.35286 (15)	0.58540 (13)	0.0472 (8)	
H99A	0.4428	0.3369	0.6042	0.057*	
H99B	0.4844	0.3920	0.5826	0.057*	
C100	0.5745 (3)	0.34145 (17)	0.61745 (14)	0.0529 (9)	
H10A	0.5628	0.3490	0.6567	0.064*	
H10B	0.5876	0.3028	0.6142	0.064*	
O62	0.64926 (18)	0.37071 (11)	0.60145 (10)	0.0548 (6)	
C101	0.6892 (2)	0.35283 (17)	0.55170 (15)	0.0539 (9)	
H10C	0.7373	0.3776	0.5426	0.065*	
H10D	0.6460	0.3551	0.5216	0.065*	
C102	0.7233 (3)	0.29830 (18)	0.55357 (14)	0.0506 (9)	
C103	0.7572 (3)	0.25520 (17)	0.55301 (12)	0.0481 (8)	
C104	0.8004 (3)	0.20284 (18)	0.55069 (16)	0.0569 (10)	
H10E	0.7564	0.1742	0.5467	0.068*	
H10F	0.8393	0.2015	0.5183	0.068*	
O63	0.84946 (18)	0.19428 (11)	0.59966 (10)	0.0555 (6)	
C105	0.8953 (3)	0.14528 (16)	0.59992 (17)	0.0605 (10)	
H10G	0.8657	0.1197	0.5751	0.073*	
H10H	0.8941	0.1301	0.6375	0.073*	
C106	0.9897 (3)	0.15148 (16)	0.58159 (14)	0.0528 (9)	
H10I	1.0208	0.1767	0.6060	0.063*	
H10J	1.0198	0.1165	0.5825	0.063*	
O64	0.98647 (17)	0.17174 (11)	0.52699 (10)	0.0487 (6)	
C107	1.0597 (2)	0.17434 (13)	0.49578 (13)	0.0378 (7)	
C108	1.0480 (2)	0.19463 (12)	0.44234 (12)	0.0339 (6)	
C109	1.1185 (2)	0.19931 (14)	0.40815 (14)	0.0413 (7)	
H109	1.1107	0.2131	0.3720	0.050*	
C110	1.2012 (2)	0.18436 (16)	0.42528 (17)	0.0504 (9)	
H110	1.2492	0.1869	0.4010	0.061*	
C111	1.2117 (2)	0.16596 (16)	0.47788 (15)	0.0495 (9)	
H111	1.2682	0.1569	0.4905	0.059*	
C112	1.1416 (2)	0.16026 (15)	0.51333 (14)	0.0464 (8)	
H112	1.1501	0.1467	0.5495	0.056*	
C121	0.6253 (4)	−0.0022 (2)	0.1057 (2)	0.0868 (15)	
H20A	0.6495	−0.0380	0.1141	0.104*	
H20B	0.6708	0.0247	0.1138	0.104*	
Cl21	0.53812 (9)	0.00932 (6)	0.14749 (8)	0.0973 (5)	
Cl22	0.59952 (18)	0.00087 (8)	0.03701 (8)	0.1307 (7)	
C122	0.1203 (4)	0.4725 (3)	−0.0727 (3)	0.136 (3)	
H20C	0.1213	0.4334	−0.0793	0.163*	0.5
H20D	0.1788	0.4866	−0.0813	0.163*	0.5
H20E	0.0923	0.4401	−0.0574	0.163*	0.5
H20F	0.1695	0.4599	−0.0955	0.163*	0.5
Cl23	0.04703 (11)	0.50107 (6)	−0.11682 (8)	0.1000 (5)	
Cl24	0.0995 (5)	0.4835 (2)	−0.0065 (3)	0.166 (2)	0.5
Cl25	0.1621 (4)	0.5047 (2)	−0.0210 (2)	0.156 (2)	0.5

### Atomic displacement parameters (Å^2^)

**Table:**

	*U*^11^	*U*^22^	*U*^33^	*U*^12^	*U*^13^	*U*^23^
Ni1	0.02661 (14)	0.02566 (15)	0.02813 (14)	−0.00067 (13)	0.0002 (2)	0.00006 (14)
N1	0.0305 (13)	0.0282 (12)	0.0321 (12)	−0.0028 (10)	−0.0010 (10)	−0.0006 (10)
N2	0.0315 (13)	0.0299 (12)	0.0291 (12)	0.0012 (10)	−0.0012 (10)	0.0010 (10)
N3	0.0344 (14)	0.0287 (12)	0.0294 (11)	−0.0041 (10)	−0.0002 (11)	0.0019 (10)
N4	0.0278 (13)	0.0297 (12)	0.0279 (11)	0.0011 (10)	0.0012 (10)	−0.0003 (9)
C1	0.0278 (15)	0.0293 (15)	0.0354 (15)	−0.0002 (12)	−0.0030 (12)	0.0020 (12)
C2	0.0302 (16)	0.0356 (16)	0.058 (2)	0.0035 (14)	−0.0041 (15)	0.0017 (14)
C3	0.0319 (16)	0.0302 (15)	0.066 (2)	0.0035 (13)	−0.0055 (16)	−0.0006 (15)
C4	0.0314 (16)	0.0296 (15)	0.0453 (16)	0.0030 (12)	0.0007 (14)	0.0005 (13)
C5	0.0325 (16)	0.0279 (14)	0.0393 (15)	0.0008 (12)	−0.0012 (13)	0.0010 (12)
C6	0.0340 (16)	0.0234 (13)	0.0359 (15)	−0.0006 (11)	0.0004 (12)	−0.0023 (11)
C7	0.0357 (16)	0.0286 (14)	0.0460 (16)	−0.0025 (12)	0.0010 (14)	−0.0012 (13)
C8	0.0309 (15)	0.0325 (15)	0.0430 (16)	−0.0046 (12)	0.0009 (13)	0.0014 (13)
C9	0.0301 (15)	0.0299 (14)	0.0287 (14)	−0.0028 (12)	−0.0002 (12)	0.0002 (11)
C10	0.0309 (15)	0.0313 (15)	0.0258 (13)	−0.0003 (12)	−0.0020 (12)	0.0003 (11)
C11	0.0279 (14)	0.0314 (15)	0.0308 (14)	−0.0003 (12)	0.0019 (11)	0.0014 (12)
C12	0.0259 (14)	0.0381 (16)	0.0399 (17)	0.0007 (13)	0.0005 (12)	−0.0021 (13)
C13	0.0359 (17)	0.0301 (14)	0.0446 (16)	0.0041 (13)	0.0005 (14)	0.0016 (13)
C14	0.0333 (16)	0.0276 (14)	0.0335 (14)	0.0024 (12)	−0.0019 (12)	0.0021 (11)
C15	0.0328 (16)	0.0285 (14)	0.0347 (15)	0.0024 (12)	−0.0005 (12)	−0.0009 (11)
C16	0.0315 (15)	0.0252 (14)	0.0390 (16)	−0.0028 (11)	0.0001 (12)	0.0019 (11)
C17	0.0366 (16)	0.0280 (14)	0.0518 (18)	−0.0033 (13)	−0.0004 (14)	0.0050 (13)
C18	0.0328 (16)	0.0334 (16)	0.0488 (18)	−0.0043 (13)	−0.0018 (14)	0.0000 (13)
C19	0.0284 (15)	0.0294 (14)	0.0288 (13)	−0.0019 (11)	−0.0017 (12)	−0.0013 (11)
C20	0.0297 (15)	0.0329 (15)	0.0278 (14)	−0.0021 (12)	−0.0040 (12)	−0.0013 (11)
C21	0.0276 (15)	0.0288 (14)	0.0567 (18)	0.0006 (12)	−0.0040 (14)	−0.0033 (13)
C22	0.0386 (17)	0.0334 (16)	0.0546 (19)	0.0008 (13)	−0.0011 (15)	0.0027 (14)
C23	0.0431 (19)	0.0325 (16)	0.072 (2)	0.0043 (14)	−0.0094 (17)	0.0068 (16)
C24	0.0426 (19)	0.0292 (16)	0.077 (3)	0.0029 (14)	−0.0109 (18)	−0.0012 (16)
C25	0.0401 (18)	0.0380 (18)	0.067 (2)	0.0024 (15)	−0.0075 (17)	−0.0158 (16)
C26	0.0321 (16)	0.0386 (17)	0.0528 (18)	0.0007 (13)	−0.0051 (14)	−0.0039 (14)
Cl1	0.0684 (6)	0.0511 (5)	0.0494 (5)	0.0118 (5)	−0.0030 (4)	0.0009 (4)
Cl2	0.0534 (5)	0.0487 (5)	0.0495 (4)	0.0010 (4)	−0.0011 (4)	−0.0020 (4)
C27	0.0292 (16)	0.0316 (15)	0.0488 (16)	0.0001 (12)	0.0006 (13)	0.0025 (13)
C28	0.0373 (16)	0.0344 (16)	0.0510 (18)	0.0019 (13)	0.0018 (15)	0.0009 (14)
C29	0.0429 (19)	0.0334 (16)	0.071 (2)	0.0035 (14)	0.0082 (17)	−0.0045 (16)
C30	0.049 (2)	0.0318 (17)	0.077 (3)	0.0069 (15)	0.0106 (19)	0.0086 (17)
Cl3	0.0653 (6)	0.0467 (5)	0.0480 (4)	0.0107 (4)	−0.0017 (4)	−0.0028 (4)
Cl4	0.0551 (5)	0.0475 (4)	0.0461 (4)	0.0000 (4)	−0.0016 (4)	0.0037 (3)
C31	0.046 (2)	0.0396 (19)	0.066 (2)	0.0040 (16)	0.0001 (17)	0.0157 (16)
C32	0.0329 (16)	0.0379 (17)	0.0525 (18)	0.0024 (14)	0.0012 (14)	0.0082 (14)
C33	0.0298 (15)	0.0282 (14)	0.0314 (14)	−0.0009 (12)	0.0037 (12)	−0.0004 (11)
C34	0.0333 (16)	0.0344 (16)	0.0313 (14)	−0.0022 (12)	0.0000 (12)	−0.0014 (12)
C35	0.0338 (16)	0.0464 (18)	0.0365 (16)	0.0012 (14)	0.0070 (14)	0.0005 (13)
C36	0.0286 (16)	0.0511 (19)	0.0480 (18)	−0.0036 (14)	0.0036 (14)	−0.0012 (15)
C37	0.0317 (17)	0.0500 (19)	0.0393 (17)	−0.0047 (14)	−0.0020 (14)	0.0003 (15)
C38	0.0360 (17)	0.0445 (18)	0.0306 (15)	−0.0040 (14)	0.0000 (13)	0.0013 (13)
O1	0.0329 (12)	0.0675 (16)	0.0270 (10)	−0.0054 (11)	−0.0021 (9)	0.0016 (10)
C39	0.048 (2)	0.0472 (18)	0.0322 (15)	0.0010 (15)	0.0050 (15)	0.0035 (14)
C40	0.056 (2)	0.0483 (19)	0.0273 (15)	0.0021 (16)	−0.0045 (14)	−0.0037 (13)
O2	0.0540 (15)	0.0487 (14)	0.0504 (14)	0.0042 (12)	−0.0146 (12)	−0.0012 (11)
C41	0.049 (2)	0.069 (3)	0.053 (2)	0.0094 (19)	−0.0082 (19)	−0.0172 (19)
C42	0.041 (2)	0.071 (3)	0.0429 (19)	0.0092 (18)	−0.0032 (16)	−0.0017 (19)
C43	0.050 (2)	0.070 (3)	0.0416 (18)	0.013 (2)	0.0034 (17)	0.0070 (18)
C44	0.049 (2)	0.068 (3)	0.053 (2)	0.0103 (19)	0.0115 (18)	0.0161 (19)
O3	0.0610 (17)	0.0506 (15)	0.0523 (15)	0.0058 (13)	0.0162 (13)	−0.0021 (11)
C45	0.065 (2)	0.055 (2)	0.0305 (16)	0.0007 (18)	0.0058 (16)	0.0042 (15)
C46	0.051 (2)	0.054 (2)	0.0262 (15)	−0.0006 (16)	−0.0028 (15)	−0.0007 (14)
O4	0.0418 (14)	0.0786 (18)	0.0282 (11)	−0.0077 (13)	−0.0014 (10)	−0.0043 (11)
C47	0.0308 (16)	0.0383 (17)	0.0344 (15)	−0.0024 (13)	−0.0020 (13)	0.0025 (12)
C48	0.0297 (15)	0.0289 (14)	0.0326 (15)	−0.0043 (12)	−0.0021 (12)	−0.0003 (12)
C49	0.0320 (17)	0.0453 (18)	0.0334 (16)	−0.0061 (14)	−0.0008 (13)	0.0008 (13)
C50	0.0315 (17)	0.052 (2)	0.0461 (19)	−0.0043 (15)	0.0019 (15)	0.0006 (16)
C51	0.0347 (17)	0.0436 (18)	0.0477 (18)	−0.0043 (14)	−0.0104 (15)	0.0031 (15)
C52	0.0443 (19)	0.0436 (18)	0.0381 (16)	−0.0058 (15)	−0.0129 (15)	0.0028 (13)
Ni2	0.02890 (15)	0.03134 (17)	0.02786 (15)	−0.00095 (15)	0.0001 (2)	−0.00108 (14)
N61	0.0312 (13)	0.0327 (13)	0.0326 (12)	−0.0005 (10)	0.0000 (11)	−0.0009 (10)
N62	0.0325 (13)	0.0349 (13)	0.0289 (12)	−0.0015 (10)	0.0002 (10)	−0.0012 (10)
N63	0.0357 (14)	0.0342 (13)	0.0284 (11)	0.0024 (11)	0.0010 (10)	−0.0017 (10)
N64	0.0288 (13)	0.0368 (13)	0.0302 (12)	−0.0035 (10)	0.0022 (10)	−0.0033 (10)
C61	0.0360 (16)	0.0350 (15)	0.0304 (15)	0.0020 (13)	−0.0007 (12)	−0.0015 (13)
C62	0.0319 (16)	0.0480 (18)	0.0408 (17)	−0.0061 (14)	−0.0013 (14)	−0.0006 (14)
C63	0.0314 (16)	0.0407 (17)	0.0502 (18)	−0.0052 (13)	0.0003 (14)	0.0030 (15)
C64	0.0389 (17)	0.0338 (16)	0.0397 (16)	−0.0069 (13)	−0.0003 (14)	0.0011 (13)
C65	0.0329 (16)	0.0385 (16)	0.0415 (16)	−0.0069 (13)	0.0011 (13)	−0.0006 (13)
C66	0.0408 (18)	0.0320 (15)	0.0368 (15)	−0.0020 (12)	−0.0002 (13)	−0.0003 (12)
C67	0.0411 (18)	0.0344 (16)	0.0465 (17)	0.0024 (14)	0.0002 (15)	−0.0011 (14)
C68	0.0412 (18)	0.0346 (16)	0.0435 (17)	0.0047 (13)	0.0005 (14)	0.0016 (13)
C69	0.0310 (15)	0.0358 (15)	0.0274 (14)	−0.0033 (12)	−0.0005 (12)	0.0025 (11)
C70	0.0347 (16)	0.0356 (15)	0.0233 (13)	0.0030 (13)	−0.0007 (12)	0.0021 (11)
C71	0.0293 (15)	0.0366 (16)	0.0275 (14)	−0.0024 (12)	0.0008 (11)	0.0004 (11)
C72	0.0335 (16)	0.0405 (16)	0.0356 (16)	−0.0030 (13)	0.0008 (13)	−0.0001 (13)
C73	0.0381 (18)	0.0402 (17)	0.0421 (17)	−0.0102 (14)	0.0026 (14)	−0.0046 (14)
C74	0.0340 (16)	0.0323 (15)	0.0317 (14)	−0.0025 (12)	0.0027 (12)	−0.0034 (12)
C75	0.0418 (18)	0.0320 (15)	0.0343 (15)	−0.0072 (13)	0.0016 (13)	−0.0031 (12)
C76	0.0347 (16)	0.0313 (14)	0.0357 (14)	−0.0011 (12)	0.0006 (13)	−0.0046 (12)
C77	0.0468 (19)	0.0352 (16)	0.0505 (18)	0.0013 (15)	0.0010 (16)	−0.0064 (15)
C78	0.0359 (17)	0.0380 (17)	0.0468 (18)	0.0072 (13)	−0.0027 (14)	−0.0063 (14)
C79	0.0335 (16)	0.0338 (15)	0.0329 (15)	0.0060 (12)	−0.0029 (13)	−0.0024 (12)
C80	0.0284 (15)	0.0452 (18)	0.0278 (14)	0.0036 (13)	0.0007 (12)	−0.0042 (12)
C81	0.0377 (18)	0.0328 (16)	0.073 (2)	−0.0032 (14)	0.0032 (17)	−0.0038 (16)
C82	0.062 (3)	0.058 (3)	0.094 (3)	−0.016 (2)	0.011 (2)	−0.029 (2)
C83	0.063 (3)	0.062 (3)	0.155 (6)	−0.017 (2)	0.010 (3)	−0.048 (4)
C84	0.062 (3)	0.040 (2)	0.185 (7)	−0.009 (2)	0.004 (4)	0.011 (4)
C85	0.049 (2)	0.045 (2)	0.155 (5)	−0.010 (2)	−0.009 (3)	0.040 (3)
C86	0.0291 (17)	0.0399 (19)	0.095 (3)	0.0001 (15)	0.0008 (17)	0.0198 (19)
Cl61	0.1178 (12)	0.1310 (13)	0.0743 (8)	−0.0583 (10)	0.0131 (8)	−0.0466 (8)
Cl62	0.0474 (5)	0.0732 (6)	0.0657 (6)	−0.0015 (5)	−0.0021 (5)	0.0268 (5)
C87	0.0345 (16)	0.0360 (16)	0.0499 (17)	−0.0016 (13)	0.0028 (14)	−0.0038 (14)
C88	0.059 (2)	0.0389 (18)	0.058 (2)	−0.0084 (16)	0.0038 (18)	0.0048 (16)
C89	0.071 (3)	0.038 (2)	0.084 (3)	−0.0072 (19)	0.008 (2)	0.008 (2)
C90	0.058 (2)	0.0344 (19)	0.110 (4)	−0.0024 (17)	0.011 (3)	−0.002 (2)
C91	0.054 (2)	0.042 (2)	0.079 (3)	−0.0036 (18)	0.004 (2)	−0.021 (2)
C92	0.0424 (19)	0.0413 (18)	0.055 (2)	−0.0025 (15)	0.0037 (16)	−0.0112 (15)
Cl63	0.1201 (10)	0.0691 (7)	0.0464 (5)	−0.0251 (7)	0.0126 (6)	0.0033 (5)
Cl64	0.0492 (5)	0.0602 (5)	0.0489 (4)	0.0002 (4)	−0.0006 (4)	−0.0139 (4)
C93	0.0306 (15)	0.0367 (16)	0.0331 (15)	−0.0004 (12)	0.0019 (13)	0.0045 (12)
C94	0.0364 (17)	0.0439 (17)	0.0342 (15)	0.0041 (14)	−0.0026 (13)	0.0020 (13)
C95	0.0433 (19)	0.060 (2)	0.0409 (17)	0.0122 (16)	0.0105 (15)	0.0013 (15)
C96	0.0353 (18)	0.071 (3)	0.053 (2)	0.0118 (17)	0.0043 (16)	0.0078 (18)
C97	0.0353 (18)	0.069 (2)	0.047 (2)	0.0100 (16)	−0.0017 (15)	0.0061 (18)
C98	0.0418 (18)	0.057 (2)	0.0311 (15)	0.0029 (16)	−0.0049 (14)	0.0085 (14)
O61	0.0384 (12)	0.0593 (14)	0.0327 (11)	0.0041 (11)	0.0005 (10)	−0.0058 (10)
C99	0.054 (2)	0.056 (2)	0.0310 (16)	0.0037 (17)	0.0045 (15)	−0.0089 (15)
C100	0.057 (2)	0.066 (2)	0.0358 (17)	−0.0009 (19)	−0.0016 (16)	−0.0014 (16)
O62	0.0592 (15)	0.0583 (15)	0.0471 (13)	−0.0015 (13)	−0.0071 (12)	−0.0010 (11)
C101	0.0447 (18)	0.069 (2)	0.0479 (19)	−0.0006 (18)	−0.0045 (16)	0.0073 (18)
C102	0.046 (2)	0.070 (3)	0.0358 (17)	−0.0089 (18)	−0.0041 (15)	0.0017 (17)
C103	0.0443 (18)	0.066 (2)	0.0344 (14)	−0.0081 (18)	0.0029 (17)	−0.0016 (16)
C104	0.055 (2)	0.074 (3)	0.0412 (19)	−0.007 (2)	0.0041 (17)	−0.0115 (18)
O63	0.0660 (16)	0.0584 (15)	0.0421 (12)	0.0038 (13)	0.0105 (12)	−0.0004 (11)
C105	0.079 (3)	0.053 (2)	0.050 (2)	0.010 (2)	0.018 (2)	0.0134 (17)
C106	0.066 (2)	0.059 (2)	0.0336 (17)	0.0074 (19)	0.0075 (16)	0.0084 (15)
O64	0.0436 (14)	0.0672 (16)	0.0354 (12)	0.0093 (12)	0.0052 (10)	0.0085 (11)
C107	0.0373 (17)	0.0414 (17)	0.0347 (16)	0.0047 (14)	−0.0015 (13)	−0.0036 (13)
C108	0.0306 (15)	0.0394 (16)	0.0316 (15)	0.0042 (13)	−0.0020 (12)	−0.0037 (12)
C109	0.0353 (17)	0.054 (2)	0.0349 (16)	0.0054 (14)	−0.0027 (14)	−0.0060 (14)
C110	0.0337 (18)	0.066 (2)	0.052 (2)	0.0043 (16)	0.0013 (15)	−0.0114 (17)
C111	0.0388 (18)	0.060 (2)	0.050 (2)	0.0091 (16)	−0.0118 (16)	−0.0092 (17)
C112	0.045 (2)	0.056 (2)	0.0381 (16)	0.0104 (16)	−0.0074 (15)	−0.0026 (15)
C121	0.074 (3)	0.090 (4)	0.096 (4)	0.009 (3)	0.000 (3)	0.020 (3)
Cl21	0.0762 (8)	0.0775 (8)	0.1383 (12)	0.0179 (7)	0.0173 (9)	0.0356 (9)
Cl22	0.189 (2)	0.1036 (12)	0.0993 (11)	−0.0046 (13)	−0.0029 (13)	0.0029 (10)
C122	0.061 (3)	0.141 (6)	0.206 (7)	0.017 (4)	−0.023 (4)	−0.079 (6)
Cl23	0.1019 (10)	0.0642 (7)	0.1339 (13)	0.0049 (7)	0.0046 (10)	−0.0104 (8)
Cl24	0.209 (6)	0.115 (4)	0.175 (4)	0.014 (4)	−0.057 (5)	−0.055 (3)
Cl25	0.179 (5)	0.119 (3)	0.170 (4)	−0.005 (3)	−0.054 (4)	−0.063 (3)

### Geometric parameters (Å, º)

**Table:**

Ni1—N4	1.937 (2)	N62—C69	1.373 (4)
Ni1—N2	1.942 (2)	N62—C66	1.387 (4)
Ni1—N1	1.943 (3)	N63—C74	1.381 (4)
Ni1—N3	1.946 (3)	N63—C71	1.389 (4)
N1—C4	1.381 (4)	N64—C76	1.386 (4)
N1—C1	1.388 (4)	N64—C79	1.405 (4)
N2—C6	1.380 (4)	C61—C80	1.383 (4)
N2—C9	1.390 (4)	C61—C62	1.431 (5)
N3—C14	1.381 (4)	C62—C63	1.336 (5)
N3—C11	1.388 (4)	C62—H62	0.9500
N4—C16	1.388 (4)	C63—C64	1.440 (5)
N4—C19	1.393 (4)	C63—H63	0.9500
C1—C20	1.369 (4)	C64—C65	1.371 (5)
C1—C2	1.437 (4)	C65—C66	1.388 (5)
C2—C3	1.344 (5)	C65—C81	1.491 (5)
C2—H2	0.9500	C66—C67	1.417 (4)
C3—C4	1.439 (5)	C67—C68	1.349 (5)
C3—H3	0.9500	C67—H67	0.9500
C4—C5	1.367 (4)	C68—C69	1.436 (4)
C5—C6	1.383 (4)	C68—H68	0.9500
C5—C21	1.498 (4)	C69—C70	1.388 (4)
C6—C7	1.432 (4)	C70—C71	1.389 (4)
C7—C8	1.347 (5)	C70—C93	1.494 (4)
C7—H7	0.9500	C71—C72	1.435 (4)
C8—C9	1.419 (4)	C72—C73	1.339 (5)
C8—H8	0.9500	C72—H72	0.9500
C9—C10	1.386 (4)	C73—C74	1.431 (5)
C10—C11	1.380 (4)	C73—H73	0.9500
C10—C33	1.490 (4)	C74—C75	1.375 (5)
C11—C12	1.432 (4)	C75—C76	1.386 (5)
C12—C13	1.334 (5)	C75—C87	1.499 (4)
C12—H12	0.9500	C76—C77	1.421 (5)
C13—C14	1.434 (5)	C77—C78	1.339 (5)
C13—H13	0.9500	C77—H77	0.9500
C14—C15	1.375 (4)	C78—C79	1.436 (4)
C15—C16	1.379 (4)	C78—H78	0.9500
C15—C27	1.491 (4)	C79—C80	1.368 (5)
C16—C17	1.422 (4)	C80—C108	1.501 (4)
C17—C18	1.349 (5)	C81—C86	1.389 (6)
C17—H17	0.9500	C81—C82	1.401 (6)
C18—C19	1.429 (4)	C82—C83	1.427 (7)
C18—H18	0.9500	C82—Cl61	1.727 (6)
C19—C20	1.374 (4)	C83—C84	1.357 (9)
C20—C48	1.489 (4)	C83—H83	0.9500
C21—C26	1.393 (5)	C84—C85	1.358 (9)
C21—C22	1.394 (5)	C84—H84	0.9500
C22—C23	1.397 (5)	C85—C86	1.390 (6)
C22—Cl1	1.736 (4)	C85—H85	0.9500
C23—C24	1.370 (6)	C86—Cl62	1.724 (5)
C23—H23	0.9500	C87—C92	1.393 (5)
C24—C25	1.379 (5)	C87—C88	1.399 (5)
C24—H24	0.9500	C88—C89	1.388 (5)
C25—C26	1.377 (5)	C88—Cl63	1.743 (4)
C25—H25	0.9500	C89—C90	1.352 (7)
C26—Cl2	1.737 (4)	C89—H89	0.9500
C27—C32	1.392 (5)	C90—C91	1.374 (7)
C27—C28	1.395 (5)	C90—H90	0.9500
C28—C29	1.387 (5)	C91—C92	1.393 (5)
C28—Cl3	1.734 (3)	C91—H91	0.9500
C29—C30	1.375 (6)	C92—Cl64	1.732 (4)
C29—H29	0.9500	C93—C94	1.397 (4)
C30—C31	1.382 (6)	C93—C98	1.404 (4)
C30—H30	0.9500	C94—O61	1.372 (4)
Cl4—C32	1.740 (4)	C94—C95	1.396 (5)
C31—C32	1.392 (5)	C95—C96	1.369 (5)
C31—H31	0.9500	C95—H95	0.9500
C33—C38	1.384 (4)	C96—C97	1.386 (6)
C33—C34	1.400 (4)	C96—H96	0.9500
C34—O1	1.371 (4)	C97—C98	1.385 (5)
C34—C35	1.388 (5)	C97—H97	0.9500
C35—C36	1.385 (5)	C98—H98	0.9500
C35—H35	0.9500	O61—C99	1.428 (4)
C36—C37	1.376 (5)	C99—C100	1.497 (5)
C36—H36	0.9500	C99—H99A	0.9900
C37—C38	1.395 (5)	C99—H99B	0.9900
C37—H37	0.9500	C100—O62	1.420 (5)
C38—H38	0.9500	C100—H10A	0.9900
O1—C39	1.441 (4)	C100—H10B	0.9900
C39—C40	1.513 (5)	O62—C101	1.429 (5)
C39—H39A	0.9900	C101—C102	1.460 (6)
C39—H39B	0.9900	C101—H10C	0.9900
C40—O2	1.420 (4)	C101—H10D	0.9900
C40—H40A	0.9900	C102—C103	1.197 (6)
C40—H40B	0.9900	C103—C104	1.468 (6)
O2—C41	1.430 (5)	C104—O63	1.426 (5)
C41—C42	1.473 (6)	C104—H10E	0.9900
C41—H41A	0.9900	C104—H10F	0.9900
C41—H41B	0.9900	O63—C105	1.413 (5)
C42—C43	1.189 (5)	C105—C106	1.529 (6)
C43—C44	1.461 (6)	C105—H10G	0.9900
C44—O3	1.422 (5)	C105—H10H	0.9900
C44—H44A	0.9900	C106—O64	1.421 (4)
C44—H44B	0.9900	C106—H10I	0.9900
O3—C45	1.420 (5)	C106—H10J	0.9900
C45—C46	1.495 (5)	O64—C107	1.363 (4)
C45—H45A	0.9900	C107—C112	1.378 (5)
C45—H45B	0.9900	C107—C108	1.406 (4)
C46—O4	1.423 (4)	C108—C109	1.374 (5)
C46—H46A	0.9900	C109—C110	1.391 (5)
C46—H46B	0.9900	C109—H109	0.9500
O4—C47	1.365 (4)	C110—C111	1.368 (6)
C47—C48	1.393 (4)	C110—H110	0.9500
C47—C52	1.396 (5)	C111—C112	1.390 (5)
C48—C49	1.387 (4)	C111—H111	0.9500
C49—C50	1.390 (5)	C112—H112	0.9500
C49—H49	0.9500	C121—Cl21	1.710 (6)
C50—C51	1.375 (5)	C121—Cl22	1.718 (6)
C50—H50	0.9500	C121—H20A	0.9900
C51—C52	1.376 (5)	C121—H20B	0.9900
C51—H51	0.9500	C122—Cl25	1.624 (7)
C52—H52	0.9500	C122—Cl24	1.664 (9)
Ni2—N63	1.937 (3)	C122—Cl23	1.712 (6)
Ni2—N64	1.939 (3)	C122—H20C	0.9900
Ni2—N62	1.948 (3)	C122—H20D	0.9900
Ni2—N61	1.950 (3)	C122—H20E	0.9900
N61—C64	1.381 (4)	C122—H20F	0.9900
N61—C61	1.388 (4)		
			
N4—Ni1—N2	177.92 (9)	C69—N62—C66	104.4 (3)
N4—Ni1—N1	90.12 (10)	C69—N62—Ni2	127.6 (2)
N2—Ni1—N1	89.82 (10)	C66—N62—Ni2	128.1 (2)
N4—Ni1—N3	89.77 (10)	C74—N63—C71	104.1 (3)
N2—Ni1—N3	90.35 (10)	C74—N63—Ni2	127.7 (2)
N1—Ni1—N3	178.43 (9)	C71—N63—Ni2	128.1 (2)
C4—N1—C1	105.0 (2)	C76—N64—C79	104.2 (2)
C4—N1—Ni1	127.8 (2)	C76—N64—Ni2	128.5 (2)
C1—N1—Ni1	127.05 (19)	C79—N64—Ni2	127.3 (2)
C6—N2—C9	104.2 (2)	C80—C61—N61	124.8 (3)
C6—N2—Ni1	128.0 (2)	C80—C61—C62	125.1 (3)
C9—N2—Ni1	127.8 (2)	N61—C61—C62	110.1 (3)
C14—N3—C11	104.6 (2)	C63—C62—C61	107.5 (3)
C14—N3—Ni1	128.1 (2)	C63—C62—H62	126.3
C11—N3—Ni1	127.12 (19)	C61—C62—H62	126.3
C16—N4—C19	104.5 (2)	C62—C63—C64	107.4 (3)
C16—N4—Ni1	128.0 (2)	C62—C63—H63	126.3
C19—N4—Ni1	127.5 (2)	C64—C63—H63	126.3
C20—C1—N1	125.7 (3)	C65—C64—N61	126.8 (3)
C20—C1—C2	123.9 (3)	C65—C64—C63	123.0 (3)
N1—C1—C2	110.1 (3)	N61—C64—C63	109.8 (3)
C3—C2—C1	107.3 (3)	C64—C65—C66	122.9 (3)
C3—C2—H2	126.4	C64—C65—C81	118.3 (3)
C1—C2—H2	126.4	C66—C65—C81	118.8 (3)
C2—C3—C4	107.2 (3)	N62—C66—C65	124.5 (3)
C2—C3—H3	126.4	N62—C66—C67	110.7 (3)
C4—C3—H3	126.4	C65—C66—C67	124.6 (3)
C5—C4—N1	125.8 (3)	C68—C67—C66	107.6 (3)
C5—C4—C3	123.8 (3)	C68—C67—H67	126.2
N1—C4—C3	110.3 (3)	C66—C67—H67	126.2
C4—C5—C6	122.7 (3)	C67—C68—C69	106.2 (3)
C4—C5—C21	118.7 (3)	C67—C68—H68	126.9
C6—C5—C21	118.3 (3)	C69—C68—H68	126.9
N2—C6—C5	124.9 (3)	N62—C69—C70	125.7 (3)
N2—C6—C7	111.2 (3)	N62—C69—C68	111.1 (3)
C5—C6—C7	123.5 (3)	C70—C69—C68	123.0 (3)
C8—C7—C6	106.2 (3)	C69—C70—C71	122.3 (3)
C8—C7—H7	126.9	C69—C70—C93	119.3 (3)
C6—C7—H7	126.9	C71—C70—C93	118.4 (3)
C7—C8—C9	107.9 (3)	N63—C71—C70	124.8 (3)
C7—C8—H8	126.0	N63—C71—C72	110.8 (3)
C9—C8—H8	126.0	C70—C71—C72	124.4 (3)
C10—C9—N2	125.1 (3)	C73—C72—C71	106.7 (3)
C10—C9—C8	124.5 (3)	C73—C72—H72	126.7
N2—C9—C8	110.5 (3)	C71—C72—H72	126.7
C11—C10—C9	122.5 (3)	C72—C73—C74	107.6 (3)
C11—C10—C33	117.4 (3)	C72—C73—H73	126.2
C9—C10—C33	120.1 (3)	C74—C73—H73	126.2
C10—C11—N3	125.7 (3)	C75—C74—N63	125.6 (3)
C10—C11—C12	124.1 (3)	C75—C74—C73	123.5 (3)
N3—C11—C12	110.1 (3)	N63—C74—C73	110.7 (3)
C13—C12—C11	107.6 (3)	C74—C75—C76	123.1 (3)
C13—C12—H12	126.2	C74—C75—C87	118.2 (3)
C11—C12—H12	126.2	C76—C75—C87	118.0 (3)
C12—C13—C14	107.2 (3)	N64—C76—C75	124.4 (3)
C12—C13—H13	126.4	N64—C76—C77	111.3 (3)
C14—C13—H13	126.4	C75—C76—C77	124.2 (3)
C15—C14—N3	125.5 (3)	C78—C77—C76	107.2 (3)
C15—C14—C13	124.0 (3)	C78—C77—H77	126.4
N3—C14—C13	110.5 (3)	C76—C77—H77	126.4
C14—C15—C16	122.7 (3)	C77—C78—C79	108.0 (3)
C14—C15—C27	118.8 (3)	C77—C78—H78	126.0
C16—C15—C27	118.1 (3)	C79—C78—H78	126.0
C15—C16—N4	125.0 (3)	C80—C79—N64	125.7 (3)
C15—C16—C17	124.3 (3)	C80—C79—C78	124.9 (3)
N4—C16—C17	110.4 (3)	N64—C79—C78	109.4 (3)
C18—C17—C16	107.6 (3)	C79—C80—C61	122.4 (3)
C18—C17—H17	126.2	C79—C80—C108	118.7 (3)
C16—C17—H17	126.2	C61—C80—C108	118.8 (3)
C17—C18—C19	107.1 (3)	C86—C81—C82	116.4 (4)
C17—C18—H18	126.4	C86—C81—C65	122.6 (3)
C19—C18—H18	126.4	C82—C81—C65	121.0 (4)
C20—C19—N4	125.5 (3)	C81—C82—C83	120.3 (5)
C20—C19—C18	124.3 (3)	C81—C82—Cl61	119.8 (3)
N4—C19—C18	110.2 (3)	C83—C82—Cl61	120.0 (4)
C1—C20—C19	122.4 (3)	C84—C83—C82	120.3 (5)
C1—C20—C48	118.4 (3)	C84—C83—H83	119.9
C19—C20—C48	119.2 (3)	C82—C83—H83	119.9
C26—C21—C22	116.0 (3)	C83—C84—C85	120.4 (5)
C26—C21—C5	122.5 (3)	C83—C84—H84	119.8
C22—C21—C5	121.6 (3)	C85—C84—H84	119.8
C21—C22—C23	122.3 (3)	C84—C85—C86	119.9 (6)
C21—C22—Cl1	119.8 (2)	C84—C85—H85	120.0
C23—C22—Cl1	117.9 (3)	C86—C85—H85	120.0
C24—C23—C22	119.0 (3)	C81—C86—C85	122.7 (5)
C24—C23—H23	120.5	C81—C86—Cl62	119.6 (3)
C22—C23—H23	120.5	C85—C86—Cl62	117.7 (4)
C23—C24—C25	120.7 (3)	C92—C87—C88	115.9 (3)
C23—C24—H24	119.7	C92—C87—C75	123.1 (3)
C25—C24—H24	119.7	C88—C87—C75	121.0 (3)
C26—C25—C24	119.3 (3)	C89—C88—C87	122.7 (4)
C26—C25—H25	120.4	C89—C88—Cl63	118.7 (3)
C24—C25—H25	120.4	C87—C88—Cl63	118.6 (3)
C25—C26—C21	122.8 (3)	C90—C89—C88	118.9 (4)
C25—C26—Cl2	117.9 (3)	C90—C89—H89	120.5
C21—C26—Cl2	119.3 (3)	C88—C89—H89	120.5
C32—C27—C28	115.7 (3)	C89—C90—C91	121.4 (4)
C32—C27—C15	122.7 (3)	C89—C90—H90	119.3
C28—C27—C15	121.6 (3)	C91—C90—H90	119.3
C29—C28—C27	122.9 (3)	C90—C91—C92	119.2 (4)
C29—C28—Cl3	117.9 (3)	C90—C91—H91	120.4
C27—C28—Cl3	119.2 (2)	C92—C91—H91	120.4
C30—C29—C28	119.1 (3)	C91—C92—C87	121.8 (4)
C30—C29—H29	120.5	C91—C92—Cl64	119.0 (3)
C28—C29—H29	120.5	C87—C92—Cl64	119.2 (3)
C29—C30—C31	120.7 (3)	C94—C93—C98	118.0 (3)
C29—C30—H30	119.7	C94—C93—C70	120.3 (3)
C31—C30—H30	119.7	C98—C93—C70	121.6 (3)
C30—C31—C32	118.7 (3)	O61—C94—C95	124.2 (3)
C30—C31—H31	120.6	O61—C94—C93	115.0 (3)
C32—C31—H31	120.6	C95—C94—C93	120.8 (3)
C27—C32—C31	122.9 (3)	C96—C95—C94	119.9 (3)
C27—C32—Cl4	119.0 (2)	C96—C95—H95	120.1
C31—C32—Cl4	118.1 (3)	C94—C95—H95	120.1
C38—C33—C34	118.5 (3)	C95—C96—C97	120.6 (3)
C38—C33—C10	122.6 (3)	C95—C96—H96	119.7
C34—C33—C10	118.8 (3)	C97—C96—H96	119.7
O1—C34—C35	124.6 (3)	C98—C97—C96	119.8 (4)
O1—C34—C33	114.3 (3)	C98—C97—H97	120.1
C35—C34—C33	121.0 (3)	C96—C97—H97	120.1
C36—C35—C34	119.2 (3)	C97—C98—C93	120.9 (3)
C36—C35—H35	120.4	C97—C98—H98	119.6
C34—C35—H35	120.4	C93—C98—H98	119.6
C37—C36—C35	120.7 (3)	C94—O61—C99	118.8 (3)
C37—C36—H36	119.6	O61—C99—C100	108.2 (3)
C35—C36—H36	119.6	O61—C99—H99A	110.1
C36—C37—C38	119.8 (3)	C100—C99—H99A	110.1
C36—C37—H37	120.1	O61—C99—H99B	110.1
C38—C37—H37	120.1	C100—C99—H99B	110.1
C33—C38—C37	120.8 (3)	H99A—C99—H99B	108.4
C33—C38—H38	119.6	O62—C100—C99	115.9 (3)
C37—C38—H38	119.6	O62—C100—H10A	108.3
C34—O1—C39	118.5 (3)	C99—C100—H10A	108.3
O1—C39—C40	106.1 (3)	O62—C100—H10B	108.3
O1—C39—H39A	110.5	C99—C100—H10B	108.3
C40—C39—H39A	110.5	H10A—C100—H10B	107.4
O1—C39—H39B	110.5	C100—O62—C101	114.9 (3)
C40—C39—H39B	110.5	O62—C101—C102	114.9 (3)
H39A—C39—H39B	108.7	O62—C101—H10C	108.6
O2—C40—C39	113.2 (3)	C102—C101—H10C	108.6
O2—C40—H40A	108.9	O62—C101—H10D	108.6
C39—C40—H40A	108.9	C102—C101—H10D	108.6
O2—C40—H40B	108.9	H10C—C101—H10D	107.5
C39—C40—H40B	108.9	C103—C102—C101	174.6 (4)
H40A—C40—H40B	107.7	C102—C103—C104	178.1 (4)
C40—O2—C41	115.1 (3)	O63—C104—C103	110.0 (3)
O2—C41—C42	114.3 (3)	O63—C104—H10E	109.7
O2—C41—H41A	108.7	C103—C104—H10E	109.7
C42—C41—H41A	108.7	O63—C104—H10F	109.7
O2—C41—H41B	108.7	C103—C104—H10F	109.7
C42—C41—H41B	108.7	H10E—C104—H10F	108.2
H41A—C41—H41B	107.6	C105—O63—C104	113.5 (3)
C43—C42—C41	177.5 (4)	O63—C105—C106	112.8 (3)
C42—C43—C44	178.0 (5)	O63—C105—H10G	109.0
O3—C44—C43	114.9 (3)	C106—C105—H10G	109.0
O3—C44—H44A	108.5	O63—C105—H10H	109.0
C43—C44—H44A	108.5	C106—C105—H10H	109.0
O3—C44—H44B	108.5	H10G—C105—H10H	107.8
C43—C44—H44B	108.5	O64—C106—C105	106.0 (3)
H44A—C44—H44B	107.5	O64—C106—H10I	110.5
C45—O3—C44	114.7 (3)	C105—C106—H10I	110.5
O3—C45—C46	114.2 (3)	O64—C106—H10J	110.5
O3—C45—H45A	108.7	C105—C106—H10J	110.5
C46—C45—H45A	108.7	H10I—C106—H10J	108.7
O3—C45—H45B	108.7	C107—O64—C106	120.6 (3)
C46—C45—H45B	108.7	O64—C107—C112	125.1 (3)
H45A—C45—H45B	107.6	O64—C107—C108	115.2 (3)
O4—C46—C45	107.1 (3)	C112—C107—C108	119.7 (3)
O4—C46—H46A	110.3	C109—C108—C107	119.2 (3)
C45—C46—H46A	110.3	C109—C108—C80	122.8 (3)
O4—C46—H46B	110.3	C107—C108—C80	117.9 (3)
C45—C46—H46B	110.3	C108—C109—C110	121.4 (3)
H46A—C46—H46B	108.5	C108—C109—H109	119.3
C47—O4—C46	120.0 (3)	C110—C109—H109	119.3
O4—C47—C48	115.3 (3)	C111—C110—C109	118.5 (4)
O4—C47—C52	124.7 (3)	C111—C110—H110	120.7
C48—C47—C52	120.0 (3)	C109—C110—H110	120.7
C49—C48—C47	118.4 (3)	C110—C111—C112	121.4 (3)
C49—C48—C20	123.0 (3)	C110—C111—H111	119.3
C47—C48—C20	118.4 (3)	C112—C111—H111	119.3
C48—C49—C50	121.9 (3)	C107—C112—C111	119.6 (3)
C48—C49—H49	119.0	C107—C112—H112	120.2
C50—C49—H49	119.0	C111—C112—H112	120.2
C51—C50—C49	118.5 (3)	Cl21—C121—Cl22	112.8 (3)
C51—C50—H50	120.7	Cl21—C121—H20A	109.0
C49—C50—H50	120.7	Cl22—C121—H20A	109.0
C50—C51—C52	121.1 (3)	Cl21—C121—H20B	109.0
C50—C51—H51	119.4	Cl22—C121—H20B	109.0
C52—C51—H51	119.4	H20A—C121—H20B	107.8
C51—C52—C47	120.0 (3)	Cl25—C122—Cl23	122.6 (5)
C51—C52—H52	120.0	Cl24—C122—Cl23	114.1 (4)
C47—C52—H52	120.0	Cl24—C122—H20C	108.7
N63—Ni2—N64	90.11 (10)	Cl23—C122—H20C	108.7
N63—Ni2—N62	89.96 (11)	Cl24—C122—H20D	108.7
N64—Ni2—N62	179.09 (9)	Cl23—C122—H20D	108.7
N63—Ni2—N61	178.44 (9)	H20C—C122—H20D	107.6
N64—Ni2—N61	89.59 (10)	Cl25—C122—H20E	106.7
N62—Ni2—N61	90.37 (10)	Cl23—C122—H20E	106.7
C64—N61—C61	105.1 (3)	Cl25—C122—H20F	106.7
C64—N61—Ni2	126.6 (2)	Cl23—C122—H20F	106.7
C61—N61—Ni2	128.3 (2)	H20E—C122—H20F	106.6

### Hydrogen-bond geometry (Å, º)

**Table:**

*D*—H···*A*	*D*—H	H···*A*	*D*···*A*	*D*—H···*A*
C3—H3···Cl62^i^	0.95	2.86	3.566 (4)	132
C13—H13···Cl64	0.95	2.89	3.632 (3)	136
C31—H31···Cl63^ii^	0.95	2.95	3.878 (4)	165
C41—H41*A*···Cl1	0.99	2.94	3.918 (4)	169
C41—H41*B*···O1	0.99	2.39	3.037 (5)	122
C44—H44*A*···Cl3	0.99	2.91	3.867 (4)	163
C44—H44*B*···O4	0.99	2.37	3.029 (5)	123
C63—H63···Cl2^iii^	0.95	2.87	3.669 (3)	142
C73—H73···Cl4	0.95	2.83	3.639 (3)	143
C101—H10*C*···Cl61	0.99	2.75	3.734 (4)	172
C101—H10*D*···O61	0.99	2.30	2.962 (4)	123
C104—H10*F*···N64	0.99	2.67	3.410 (5)	132
C104—H10*F*···O64	0.99	2.40	3.028 (5)	121
C121—H20*B*···O62^iv^	0.99	2.65	3.304 (7)	124
C121—H20*A*···Cl2^v^	0.99	2.90	3.563 (6)	125
C122—H20*F*···Cl4^iv^	0.99	2.70	3.583 (6)	149

Symmetry codes: (i) *x*−1, *y*, *z*; (ii) −*x*+1, −*y*, −*z*+1; (iii) *x*+1, *y*, *z*; (iv) *x*, −*y*+1/2, *z*−1/2; (v) −*x*+1, *y*−1/2, −*z*+1/2.

## References

[bb1] Andrioletti, B., Ricard, D. & Boitrel, B. (1999). *New J. Chem.* **23**, 1143–1150.

[bb2] Battersby, A. R. & Hamilton, A. D. (1980). *J. Chem. Soc. Chem. Commun.* pp. 117–119.

[bb3] Brandenburg, K. (2014). *DIAMOND*. Crystal Impact GbR, Bonn, Germany.

[bb4] Collman, J. P., Lee, V. J., Kellen-Yuen, C. J., Zhang, X., Ibers, J. A. & Brauman, J. I. (1995). *J. Am. Chem. Soc.* **117**, 692–703.

[bb5] Dommaschk, M., Näther, C. & Herges, R. (2015*a*). *J. Org. Chem.* **80**, 8496–8500. 10.1021/acs.joc.5b01524 26301895

[bb6] Dommaschk, M., Peters, M., Gutzeit, F., Schütt, C., Näther, C., Sönnichsen, F. D., Tiwari, S., Riedel, C., Boretius, S. & Herges, R. (2015*b*). *J. Am. Chem. Soc.* **137**, 7552–7555. 10.1021/jacs.5b00929 25914182

[bb7] Ganesh, K. N. & Sanders, J. K. M. (1980). *J. Chem. Soc. Chem. Commun.* pp. 1129–1131.

[bb8] Gehrold, A. C., Bruhn, T., Schneider, H., Radius, U. & Bringmann, G. (2015). *Org. Lett.* **17**, 210–213. 10.1021/ol503286s 25556288

[bb9] Goncalves, D. P. N. & Sanders, J. K. M. (2007). *Synlett*, **4**, 591–594.

[bb10] Groom, C. R., Bruno, I. J., Lightfoot, M. P. & Ward, S. C. (2016). *Acta Cryst.* B**72**, 171–179. 10.1107/S2052520616003954 PMC4822653 27048719

[bb11] Gunter, M. J., Hockless, D. C. R., Johnston, M. R., Skelton, B. W. & White, A. H. (1994). *J. Am. Chem. Soc.* **116**, 4810–4823.

[bb12] Gunter, M. J., Jeynes, T. P. & Turner, P. (2004). *Eur. J. Org. Chem.* pp. 193–208.

[bb13] Koepf, M., Trabolsi, A., Elhabiri, M., Wytko, J. A., Paul, D., Albrecht-Gary, A. M. & Weiss, J. (2005). *Org. Lett.* **7**, 1279–1282. 10.1021/ol050033p 15787486

[bb14] Littler, B. J., Miller, M. A., Hung, C. A., Wagner, R. W., O’Shea, D. F., Boyle, P. D. & Lindsey, J. S. (1999). *J. Org. Chem.* **64**, 1391–1396.

[bb15] Liu, Q., Tang, M., Zeng, W., Zhang, X., Wang, J. & Zhou, Z. (2016). *Eur. J. Inorg. Chem.* pp. 5222–5229.

[bb16] Momenteau, M., Mispelter, J., Loock, B. & Bisagni, E. (1983). *J. Chem. Soc. Perkin Trans. 1*, pp. 189–196.

[bb17] Morgan, B. & Dolphin, D. (1987). *Struct. Bond.* **64**, 115–203.

[bb18] Peters, M. K., Hamer, S., Jäkel, T., Röhricht, F., Sönnichsen, F. D., von Essen, C., Lahtinen, M., Naether, C., Rissanen, K. & Herges, R. (2019). *Inorg. Chem.* DOI: 10.1021/acs.inorgchem.9b00349. 10.1021/acs.inorgchem.9b00349 30938518

[bb19] Peters, M. K. & Herges, R. (2018). *Inorg. Chem.* **57**, 3177–3182. 10.1021/acs.inorgchem.7b03164 29498852

[bb20] Peters, M. K., Näther, C. & Herges, R. (2018). *Acta Cryst.* E**74**, 1013–1016. 10.1107/S2056989018008605 PMC6038623 30002906

[bb21] Sabat, M. & Ibers, J. A. (1982). *J. Am. Chem. Soc.* **104**, 3715–3721.

[bb22] Shankar, S., Peters, M. K., Steinborn, K., Krahwinkel, B., Sönnichsen, F., Grote, D., Sander, W., Lohmiller, T. & Herges, R. (2018). *Nat. Commun.* **9**, 1–12. 10.1038/s41467-018-07023-1 PMC6232099 30420598

[bb23] Sheldrick, G. M. (2008). *Acta Cryst.* A**64**, 112–122. 10.1107/S0108767307043930 18156677

[bb24] Sheldrick, G. M. (2015*a*). *Acta Cryst.* A**71**, 3–8.

[bb25] Sheldrick, G. M. (2015*b*). *Acta Cryst.* C**71**, 3–8.

[bb26] Spek, A. L. (2009). *Acta Cryst.* D**65**, 148–155. 10.1107/S090744490804362X PMC2631630 19171970

[bb27] Stoe (2008). *X-AREA*, *X-RED* and *X-SHAPE*. Stoe & Cie, Darmstadt, Germany.

[bb28] Venkataramani, S., Jana, U., Dommaschk, M., Sönnichsen, F. D., Tuczek, F. & Herges, R. (2011). *Science*, **331**, 445–448. 10.1126/science.1201180 21273483

[bb29] Westrip, S. P. (2010). *J. Appl. Cryst.* **43**, 920–925.

